# Supplementary material for: Effects of human development, primary care coverage and the COVID-19 pandemic on the fertility rate among adolescents in Brazil in the period 2012–2021
Source: PLoS One. 2025 Oct 9;20(10):e0327656. doi: 10.1371/journal.pone.0327656 (PMC12510510; doi:10.1371/journal.pone.0327656)
Supplement: Attachment — Submitted filename: renamed_49436.docx [file pone.0327656.s001.docx]

Na minha visão que o artigo tenha um problema e/ou uma limitação metodológica importante que precisa ser destacada pelos autores.

No texto na descrição acerca da estimação da Taxa de Fecundidade adolescente está assim” the fertility rate per 1,000 women was the outcome variable, being calculated using the formula: number of live births (LB) in women of a given age group in the location and period of interest divided by the female population of the same age group, location and period of interest, multiplied by 1,000.” Para o cálculo desse indicador, central para o estudo, destaco dois pontos que merecem melhor detalhamento e importância no texto.

1) É necessário que os autores indiquem os problemas que devem ser discutidos no tanto no que diz respeito ao numerados quanto ao denominador. O número de nascimentos sofre do problema de subregistro, especialmente para as regiões Norte e Nordeste. Como os autores lidaram com esse problema? O número de meninas de 10 a 14 e de 15 a 19, por serem oriundos da projeção também apresenta problemas, em especial depois da divulgação dos resultados do Censo de 2022 e a retroprojeção divulgada pelo IBGE, que corrigiu as estimativas passadas. Esses números estavam muito sobrerepresentados para a maior parte do país, o que tornava as taxas pouco consistentes em todos os casos. É o que os autores mostram para o estado de Roraima, em que apontam para a elevada imigração de Venezuelanos. Essas informações fazem toda diferença na hora da comparabilidade com outros estudos já existentes.
2) Ainda sobre a estimativa das taxas de fecundidade, é necessário que os autores indiquem a metodologia adotada para a estimativa nos municípios. Isso porque diversos estudos apontam para a inviabilidade de usar os dados diretos, uma vez que se tratam de pequenos números e estes podem sofrer diversos efeitos que prejudicam as análises. O que foram feitos para os casos que em um determinado ano não havia registro de nascimento para meninas nos grupos analisados? Ou os números eram muito baixos? Será que esse dado era de fato o real ou sofreu o efeito de algum evento atípico, subenumeração, etc.? A estimativa para pequenas áreas merece maior detalhamento.
3) Sobre os dados para Roraima, acho que também cabe a discussão da população indígena e o aumento do número de garimpeiros e conflitos na região, que contribuíram para o aumento dos casos de violência sexual contra meninas. Como vocês teriam os dados municipais, seria interessante mostrar as diferenças daqueles de fronteira.

De modo geral achei a questão dos municípios pouco explorada nos dados, tinha a expectativa de ver mais citações de alguns exemplos com a análise de alguns municípios em destaque. Acho que isso potencializaria os resultados do paper.
